# LncRNA XR_596701 protects H9c2 cells against intermittent hypoxia-induced injury through regulation of the miR-344b-5p/FAIM3 axis

**DOI:** 10.1038/s41420-022-00834-8

**Published:** 2022-01-28

**Authors:** Qingshi Chen, Guofu Lin, Lanlan Lin, Jiefeng Huang, Lida Chen, Ningfang Lian, Mengxue Chen, Aiming Zeng, Qichang Lin

**Affiliations:** 1 Department of Endocrinology and Metabolism, The Second Affiliated Hospital of Fujian Medical University, No. 950 Donghai Street, Fengze District, Quanzhou, 362000 China; 2 Department of Respiratory and Critical Care Medicine, The Second Affiliated Hospital of Fujian Medical University, No. 34 Zhongshan North Road, Licheng District, Quanzhou, 362000 China; 3 Department of Respiratory and Critical Care Medicine, The First Affiliated Hospital of Fujian Medical University, No. 20 Chazhong Road, Taijiang District, Fuzhou, 350005 China; 4 Department of Respiratory and Critical Care Medicine, Zhangzhou Affiliated Hospital of Fujian Medical University, No. 59, Shenglixi Road, Xiangcheng District, Zhangzhou, 363000 China

**Keywords:** Cell death, Molecular biology

## Abstract

Long noncoding RNAs (lncRNAs) participate in various biological processes and cardiovascular diseases. Recently, a novel lncRNA XR_596701 was found to be differentially expressed in obstructive sleep apnea (OSA)-induced myocardial tissue compared to normal myocardial tissues. However, the pathological effect and regulatory mechanism of XR_596701 in intermittent hypoxia (IH)-mediated cardiomyocytes damage have not been studied. The subcellular localization of XR_596701 was determined by fluorescence in situ hybridization (FISH). Gene expressions of XR_596701 and miR-344b-5p were detected by quantitative real-time polymerase chain reaction (qRT-PCR) in IH-induced H9c2 cells. Cell proliferation was measured by 5-ethynyl-2′-deoxyuridine (EdU) staining assay. Cell apoptosis was detected by Hoechst 33342/PI staining and immunofluorescence (IF). Apoptotic protein of H9c2 cells was measured by western blot. The direct interaction between XR_596701 and miR-344b-5p as well as miR-344b-5p and Fas apoptotic inhibitory molecule 3 (FAIM3) were examined using dual-luciferase reporter assay. The significance of XR_596701 and miR-344b-5p on cell proliferation and apoptosis was evaluated by using gain-of-function and loss-of-function approaches. XR_596701 was upregulated, while miR-344b-5p downregulated in IH-induced H9c2 cells. Functionally, suppression of XR_596701 and overexpression of miR-344b-5p inhibited cell proliferation and promoted cell apoptosis in H9c2 cells. The roles of XR_596701 were achieved by sponging miR-344b-5p. And the function of miR-344b-5p was reversed by targeting FAIM3. Additionally, FAIM3 mediated IH-induced H9c2 cells damage by XR_596701. XR_596701 was serve as a novel lncRNA that indicated protective roles on proliferation and apoptosis of IH-induced H9c2 cells through the miR-344b-5p/FAIM3 axis.

## Introduction

Obstructive sleep apnea (OSA), a common sleep disorder, is characterized by recurrent episodes of upper airway collapse during sleep, leading to recurrent events of nocturnal hypoxemia, hypercapnia and transient awakening [1]. Until now, several studies have focused on the relationship between OSA and cardiovascular (CV) diseases. Clinical studies have found that the prevalence of OSA in patients with coronary heart disease ranged from 30 to 58% and approximately 70% male hospitalized patients with myocardial infarction (MI) also have OSA [2, 3]. Furthermore, OSA has been recognized a major risk factor for CV diseases [4]. Although it is likely that OSA negatively affects the pathological process of heart diseases through multiple complex mechanisms such as endothelial dysfunction and inflammation [5, 6], intermittent hypoxia (IH) in particular is the important player in OSA-associated CV diseases [7]. However, the potential mechanism between IH and CV disease is largely unknown.

Recent year, long noncoding RNAs (lncRNAs) are considered as the critical noncoding RNAs that transcript longer than 200 nucleotides [8]. Emerging evidence supports that lncRNAs can exert vital roles in cardiac remodeling including MI, proliferation, and apoptosis of cardiomyocyte and myocardial fibrosis [9–11]. For example, overexpression of anti-apoptotic lncRNA Sarrah showed a better recovery of cardiac contractile function in an acute MI model mice compared to normal mice [10]. Downregulation of lncRNA myocardial infarction-associated transcript (MIAT) significantly alleviated atrial fibrillation (AF) and AF-induced myocardial fibrosis by targeting miR-133a-3p [12]. Likewise, miRNAs as short noncoding RNA molecules also exert crucial effects in abnormal cardiac physiology, particularly concentrating on cardiac hypertrophy, cardiac fibrosis and ischemic heart disease [13, 14]. To date, a numbers of studies focused on the roles of lncRNAs or miRNAs in the potential association between hypoxia/reperfusion (H/R) and cardiac injury [15]. However, the biological role and regulatory mechanism of lncRNAs in IH-mediated myocardial damage have not been studied.

Our previous study has found that several abnormally expressed lncRNAs in a rat myocardial injury model of IH were identified by using lncRNA microarray experiments and qRT-PCR. Results demonstrated that lncRNA XR_596701 was markedly elevated in the IH-induced myocardial tissue [16]. However, the expression level, biological function and molecular mechanism of XR_596701 in vitro model of myocardial injury are still unclear. Therefore, this study aimed to explore the effect of XR_596701 in IH-induced H9c2 cells and underlying molecular mechanism of XR_596701.

## Results

### Molecular characteristics and cellular localization of Rat-derived XR_596701

**Fig. 1:**
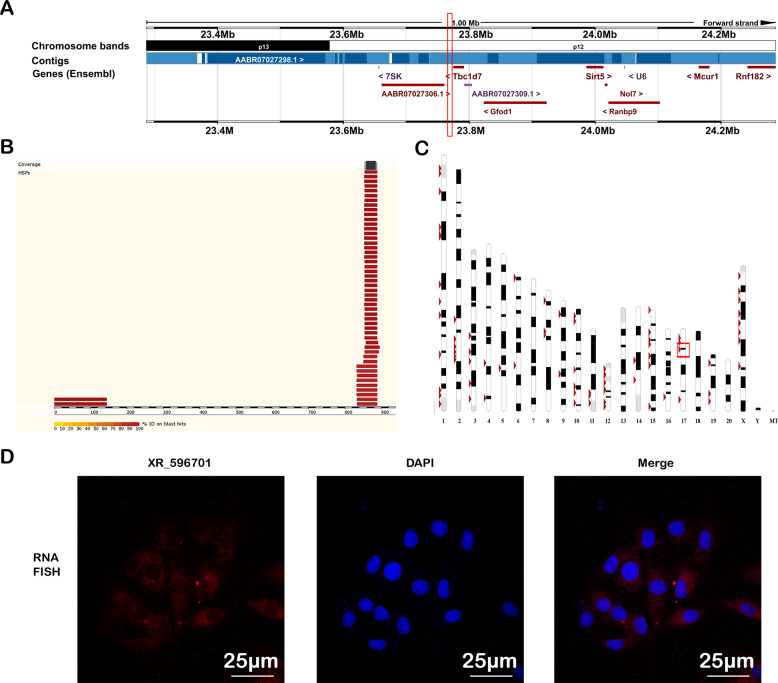
Characterization of the lncRNA XR_596701 sequence. **A** Detailed overview of the genome region. The gene colors are as follows: blue, and purple genes: non-coding; red genes: protein coding; the red squares indicate the location of XR_596701. **B** High-scoring segment pair (HSP) is a subsegment of a pair of sequences that share high level of similarity. Fragments of the query sequence that hit other places in the genome are shown as red boxes. **C** The level of similarity between the sequences depends on the sequences, the alignment algorithm, and the settings used, one can find numerous HSPs within a given pair of inputted sequences. **D** The FISH analysis of XR_596701 in H9c2 cells. XR_596701 is shown in red, and DAPI is shown in blue to indicate the cell nucleus, scale bar 25 µm. XR_596701 predominantly is distributed in the cytoplasmic region in H9c2 cells.

To explore the biologic characteristics of XR_596701, 938 bp of the full-length nucleotide sequence of XR_596701 was obtained by 5′ and 3′ RACE (Table S2). The full length of XR_596701 was then used in a BLAST search with the genomic sequences of rat for homology analysis, For the rat alignment, the sequence at genomic location 17:23774793–23775588 scored 1573 with an *e*-val of 0, which suggests that XR_596701 is located in 17:23774793–23775588 (Fig. 1A). Furthermore, analysis based on high-scoring segment pair (HSP) distribution of genome (Fig. 1B) and HSP distribution of query sequence (Fig. 1C) demonstrated that XR_596701 is located on chromosome 17 in rat. The ORF of XR_596701 was predicted by NCBI ORF Finder and further validated the coding capacity by SmartBLAST. The results indicated that the ORF has no coding capacity in rat genome (Table S3). In addition, we also assessed the protein-coding potential of XR_596701 by using CPC, the results indicated that the gene XR_596701 has a very low coding potential (Table S4).

To determine the cellular localization of the XR_596701 transcript, the expression of XR_596701 in subcellular locations was measured. The FISH analysis of XR_596701 in H9c2 cells indicated that XR_596701 is predominantly located in the cytoplasm (Fig. 1D). As we know, the function of lncRNA depends on its subcellular distribution [17], this finding will provide the evidence that XR_596701 might serve as an endogenous RNA (ceRNA) to affect the expression of its target miRNA or mRNA in cytoplasm.

### XR_596701 was upregulated and played a protective role in IH-induced H9c2 cells injury

**Fig. 2:**
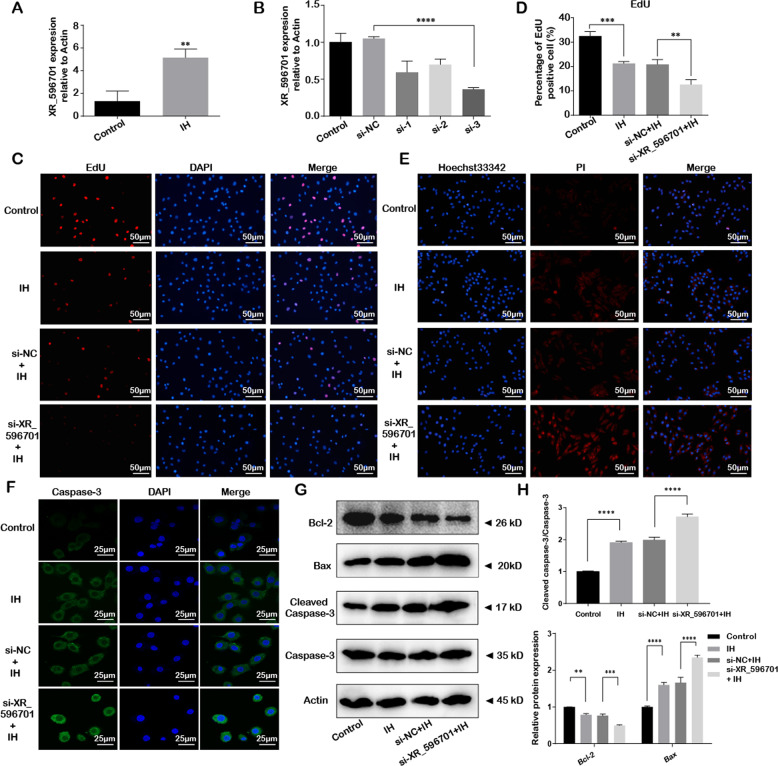
The expression level of XR_596701 and its protective role in IH-induced H9c2 cells injury. **A** The expression of XR_596701 was detected by qRT-PCR after IH stimulation, Actin mRNA served as an internal control. **B** H9c2 cells were transfected with siRNAs of XR_596701 or control siRNA (si-NC). Relative XR_596701 expression was measure by qRT-PCR. **C**, **D** H9c2 cells were transfected with si- XR_596701 or si-NC and were treated with IH for six cycles. EdU assay was performed to assess the impact of XR_596701 on the proliferation of H9c2 cells. Red (EdU) H9c2 cells indicated proliferating the cell nucleus and blue (DAPI) represented the cell nucleus, scale bar 50 µm. **E** H9c2 cells were transfected with si- XR_596701 or si-NC and were treated with IH for six cycles. Hoechst 33342/PI staining was performed to assess the efffect of XR_596701 on the apoptosis of H9c2 cells. Red (PI) indicated apoptotic H9c2 cells and blue (Hoechst 33342) represented the cell nucleus, scale bar 50 µm. **F** H9c2 cells were transfected with si- XR_596701 or si-NC and were treated with IH for six cycles. Caspase-3 immunofluorescence was measured to show the apoptosis of H9c2 cells. Caspase-3 is shown in green, and DAPI is shown in blue to indicate the cell nucleus. scale bar 25 µm. **G**, **H** H9c2 cells were transfected with si- XR_596701 or si-NC and were treated with IH for six cycles. Expression levels of apoptosis-related proteins (Bcl-2, Bax and Cleaved caspase-3/Caspase-3) by western blot analysis. *P < 0.05, **P < 0.001, ***P < 0.0001, ****P < 0.00001. Data were shown as Mean ± SD based on three independent experiments.

To determine the potential functional effects of XR_596701, H9c2 cells were treated with IH for six cycles, and the total RNA of H9c2 cells was extracted for qRT-PCR. The result showed that XR_596701 was upregulated under IH condition compared to the non-treated cells (*P* < 0.01; Fig. 2A). Simultaneously, we performed the loss-of-function experiment by knocking down XR_596701 with si-XR_596701 (Fig. 2B). First of all, our data showed that IH exposure significantly decreased cells proliferation and increased cells apoptosis when compared to the non-treated group, which is consistent with the result of our previous study [18]. After down-regulation of XR_596701, we found that si-XR_596701 not only significantly decreased cells proliferation, but also promoted cells apoptosis in IH-treated H9c2 cells model when compared to si-NC group. As shown in the Fig. 2C, D, an EdU-594 staining assay was utilized to show the cells proliferation, the results showed that the proliferation rate of H9c2 cells in si-XR_596701 group is significantly lower than si-NC group under IH exposure. In the Fig. 2E, Hoechst/PI staining assay was conducted to evaluate the cells apoptosis, the results demonstrated that the apoptosis of H9c2 cells in si-XR_596701 group is significantly higher than si-NC group under IH condition. Meanwhile, we also performed the immunofluorescence to investigate the expression level of caspase-3, which serves as a critical executioner in the intrinsic and extrinsic apoptotic pathways [19]. As shown Fig. 2F, si-XR_596701 markedly increased caspase-3 immunofluorescence expression in IH-treated H9c2 cells when compared to si-NC group after IH stimulation. Additionally, flow cytometry results also indicated that the cell apoptotic rate in the si-XR_596701 group was markedly upregulated compared to control group (Fig. S2). To further assess the cells apoptosis, we tested the apoptosis-related proteins Bax, Caspase-3, and Bcl-2 by western blot. We found that si-XR_596701 group has lower Bax and Cleaved caspase-3/Caspase-3 ratio with a higher Bcl-2 protein expression level when compared to si-NC group after IH stimulation (Fig. 2G, H).

Moreover, we performed the gain-of-function experiment by overexpressing XR_596701 with oe-XR_596701 plasmid (Fig. S3A). The results indicated that overexpression of XR_596701 could significantly increase the proliferation of H9c2 cells (as detected by EdU-594 assay in Fig. S3B, C) and reduce the H9c2 cells from IH-mediated apoptosis (as detected by western blot for apoptosis-related protein in Fig. S3D, E) when compared to empty vector group. Together, these outcomes suggest that XR_596701 exerts a protective effect in IH-induced H9c2 cells injury.

### miR-344b-5p was downregulated and mediates IH-induced H9c2 cells injury

**Fig. 3:**
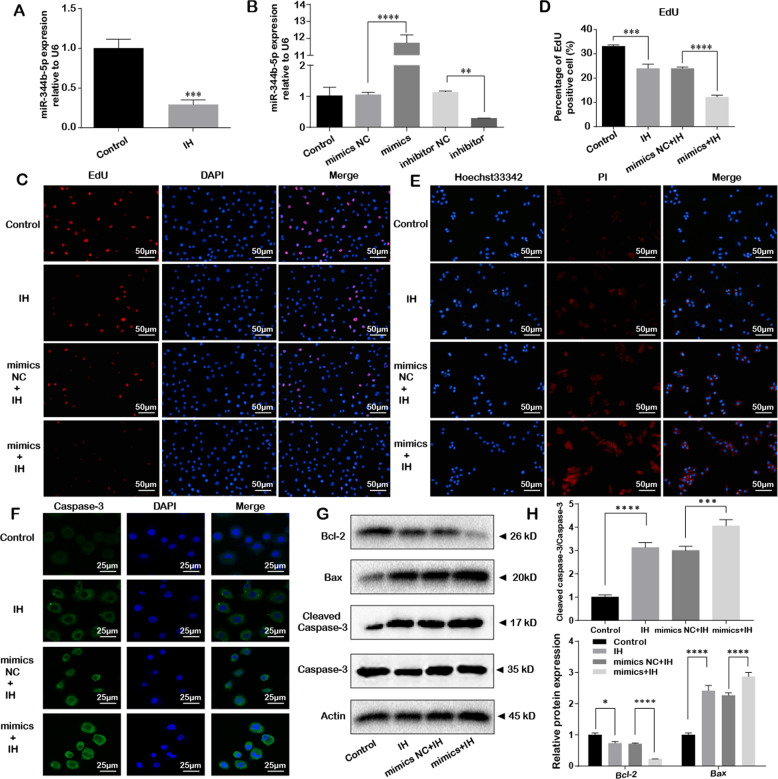
miR-344b-5p was down-regulated and mediates IH-induced cells injury. **A** The expression of miR-344b-5p was measured by qRT-PCR after IH exposure, Actin mRNA served as an internal control. **B** H9c2 cells were transfected with miR-344b-5p mimics, miR-344b-5p inhibitor, and corresponding scrambled control. Relative miR-344b-5p expression was measure by qRT-PCR. **C**, **D** H9c2 cells were transfected with miR-344b-5p mimics or mimics NC and were treated with IH for six cycles. EdU staining assay was performed to evaluate the effect of miR-344b-5p on the proliferation of H9c2 cells, scale bar 50 µm. **E** H9c2 cells were transfected with miR-344b-5p mimics or mimics NC and were treated with IH for six cycles. Hoechst 33342/PI staining was performed to evaluate the effect of miR-344b-5p on the apoptosis of H9c2 cells, scale bar 50 µm. **F** H9c2 cells were transfected with miR-344b-5p mimics or mimics NC and were treated with IH for six cycles. Caspase-3 immunofluorescence was measured to show the apoptosis of H9c2 cells, scale bar 25 µm. **G**, **H** Expression levels of apoptosis-related proteins (Bcl-2, Bax and Cleaved caspase-3/Caspase-3) by western blot. *P < 0.05, **P < 0.001, ***P < 0.0001, ****P < 0.00001. Data were shown as mean ± SD based on three independent experiments.

To further assess the effect of miR-344b-5p in H9c2 cells, we treated H9c2 cells with IH stimulation. Firstly, we examined the expression of miR-344b-5p by qRT-PCR, we found that the expression of miR-344b-5p was down-regulated under IH condition compared to the non-treated group (*P* < 0.001; Fig. 3A). Next, we applied gain-of-function strategy to examine the biological significance of miR-344b-5p on IH-induced cells injury (Fig. 3B).

After transfecting miR-344b-5p mimics or mimics NC, we found that miR-344b-5p mimics could significantly reduce the proliferation of H9c2 cells (as detected by EdU-594 assay in Fig. 3C, D) and increase the H9c2 cells from IH-induced apoptosis (as detected by Hoechst/PI assay in Fig. 3E, caspase-3 immunofluorescence in Fig. 3F and western blot for apoptosis-related protein in Fig. 3G, H) when compared to mimics NC group after IH stimulation.

### miR-344b-5p was a direct target of XR_596701 and miR-344b-5p inhibitor reversed the protective effects of XR_596701

**Fig. 4:**
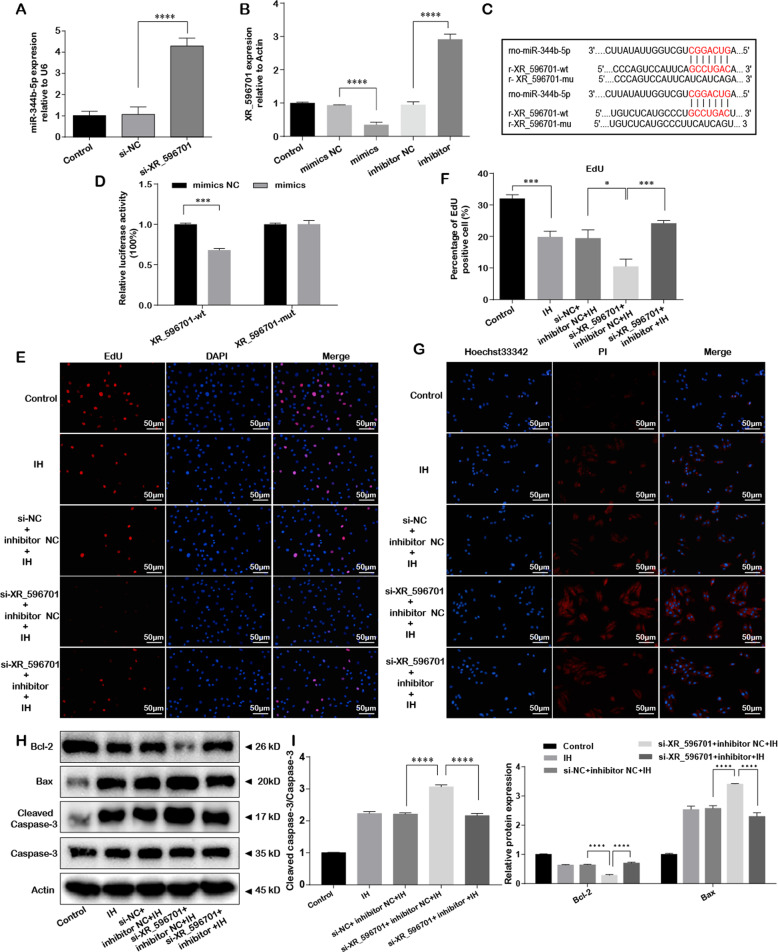
miR-344b-5p was a direct target of XR_596701 and miR-344b-5p inhibitor reversed the protective effects of XR_596701. **A** H9c2 cells were transfected with si-XR_596701 or si-NC. Relative miR-344b-5p expression was measure by qRT-PCR. **B** Relative XR_596701 expression was measure by qRT-PCR after transfecting miR-344b-5p mimics, miR-344b-5p inhibitor, and corresponding scrambled control. **C** The putative binding sites between XR_596701 and miR-344b-5p. **D** Luciferase reporter assay. Luciferase construct containing either wild-type XR_596701 (XR_596701-wt) or mutant XR_596701 (XR_596701-mut) sequence was co-transfected into H9c2 cells with miR-344b-5p mimic or mimics NC. **E**, **F** si-XR_596701 (or si-NC) and miR-344b-5p inhibitor (or inhibitor NC) were co-transfected into H9c2 cells. Cells were treated with IH for six cycles. EdU staining assay was performed to evaluate the proliferation of H9c2 cells, scale bar 50 µm. **G** Hoechst 33342/PI staining was performed to evaluate the apoptosis of H9c2 cells. si-XR_596701 (or si-NC) and miR-344b-5p inhibitor (or inhibitor NC) were co-transfected into H9c2 cells, scale bar 50 µm. Cells were treated with IH for six cycles. **H**, **I** si-XR_596701 (or si-NC) and miR-344b-5p inhibitor (or inhibitor NC) were co-transfected into H9c2 cells. Cells were treated with IH for six cycles. Expression levels of apoptosis-related proteins (Bcl-2, Bax and Cleaved caspase-3/Caspase-3) by western blot. *P < 0.05, **P < 0.001, ***P < 0.0001, ****P < 0.00001. Data were shown as mean ± SD based on three independent experiments.

Accumulating evidence indicated that lncRNAs serves as miRNA sponges in several diseases including hypoxia‐mediated injury [20]. The opposite regulatory effects of XR_596701 and miR-344b-5p in IH-induced cells injury suggested that XR_596701 may function as a sponge of miR-344b-5p. To explore the relationship between XR_596701 and miR-344b-5p, we performed qRT-PCR and the luciferase reporter assay. The results of qRT-PCR demonstrated miR-344b-5p expression was markedly increased by knocking down XR_596701 when compared to si-NC group (Fig. 4A). Analogously, and the expression level of XR_596701 significantly changed after transfecting miR-344b-5p mimics or inhibitor when compared to corresponding negative control (Fig. 4B). Additionally, the binding regions between XR_596701 and miR-344b-5p were showed in Fig. 4C. The dual-luciferase reporter indicated that the luciferase activity of wt-XR_596701 rather than mut-XR_596701 can be significantly decreased by miR-344b-5p mimics but not mimics NC (Fig. 4D). These findings supported the direct interaction between XR_596701 and miR-344b-5p.

Furthermore, we examined the effect of miR-344b-5p mimics on XR_596701-associated IH-induced cells damage. H9c2 cells were transfected with si-XR_596701, miR-344b-5p inhibitor and corresponding scramble. As shown in the Fig. 4E–I, miR-344b-5p inhibitor was able to rescue the effect of XR_596701 silence on the obvious reduction of cells proliferation, the significant increase of cells apoptosis and apoptosis-related proteins expression under IH condition. Collectively, these investigations demonstrated that directly targeting miR-344b-5p is an essential mechanism by which XR_596701 mediates H9c2 cells injury under IH stimulation.

### Fas apoptotic inhibitory molecule 3 (FAIM3) was confirmed as a direct target of miR-344b-5p and exerted antagonistic effects in miR-344b-5p mediated IH-induced cells damage

**Fig. 5:**
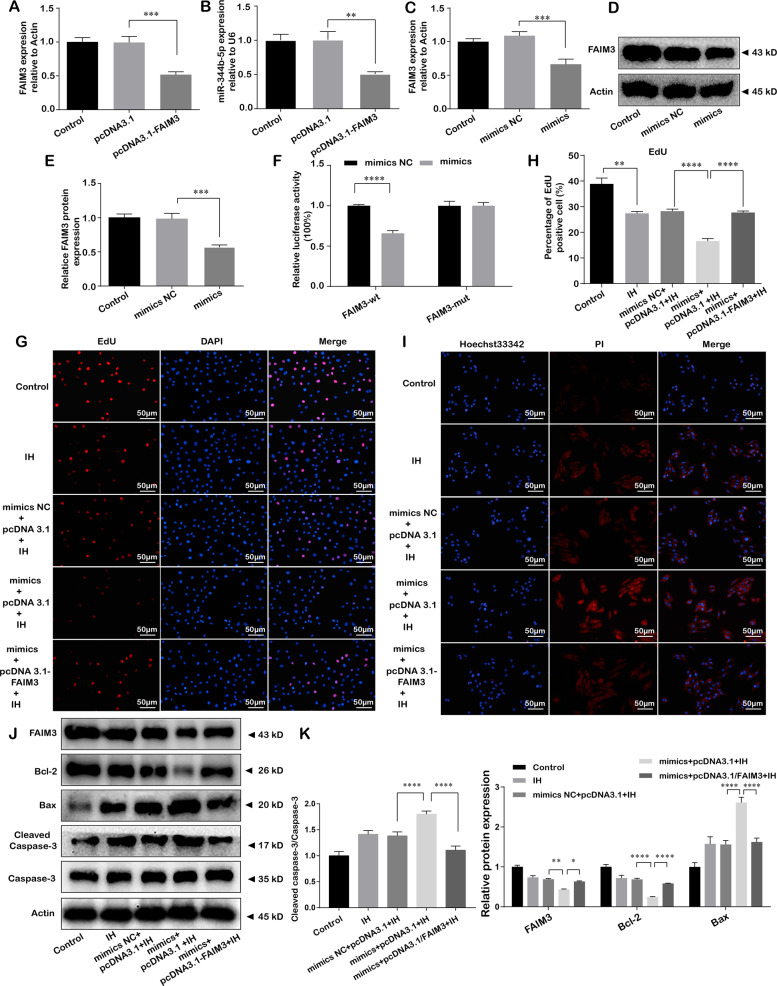
FAIM3 was confirmed as a direct target of miR-344b-5p and exerted antagonistic effects in miR-344b-5p mediated IH-induced cells damage. **A** Relative FAIM3 expression was measure by qRT-PCR after transfecting pcDNA3.1 or pcDNA3.1-FAIM3. **B** Relative miR-344b-5p expression was measure by qRT-PCR after transfecting pcDNA3.1 or pcDNA3.1-FAIM3. **C** Relative FAIM3 expression was measure by qRT-PCR after transfecting miR-344b-5p mimic or mimics NC. **D**, **E** Relative FAIM3 protein expression was measure by western blot after transfecting miR-344b-5p mimic or mimics NC. **F** Luciferase reporter assay. Cells were co-transfected with FAIM3-wt or FAIM3-mut 3′-UTR reporters and miR-344b-5p mimic or mimics NC. **G**, **H** miR-344b-5p mimic (or mimics NC) and pcDNA3.1-FAIM3 (or pcDNA3.1) were co-transfected into H9c2 cells. Cells were treated with IH for six cycles. EdU staining assay was performed to evaluate the proliferation of H9c2 cells, scale bar 50 µm. **I** Hoechst 33342/PI staining was performed to evaluate the apoptosis of H9c2 cells. miR-344b-5p mimic (or mimics NC) and pcDNA3.1-FAIM3 (or pcDNA3.1) were co-transfected into H9c2 cells, scale bar 50 µm. Cells were treated with IH for six cycles. **J**, **K** miR-344b-5p mimic (or mimics NC) and pcDNA3.1-FAIM3 (or pcDNA3.1) were co-transfected into H9c2 cells. Cells were treated with IH for six cycles. Expression levels of FAIM3 and apoptosis-related proteins (Bcl-2, Bax, and Cleaved caspase-3/Caspase-3) by western blot. *P < 0.05, **P < 0.001, ***P < 0.0001, ****P < 0.00001. Data were shown as Mean ± SD based on three independent experiments.

We predicted the potential target of miR-344b-5p by bioinformatics analysis. Using TargetScan and miRbase database, FAIM3 was predicted as a new target gene for miR-344b -5p. Binding sites between them were showed in Fig. S1. To verify the interaction between miR-344b-5p and FAIM3, we next conducted qRT-PCR and the dual-luciferase reporter assay. As shown in the Fig. 5A, B, miR-344b-5p expression was markedly decreased by pcDNA3.1-FAIM3. Similarly, the expressions of FAIM3 at mRNA and protein level were dramatically reduced by miR‐344b‐5p mimics (Fig. 5C–E). Moreover, the results of dual-luciferase reporter assay demonstrated that the luciferase activity of wt-FAIM3 was significantly decreased by miR‐344b‐5p mimics, whereas almost unchanged in the luciferase activity of mut-FAIM3 (Fig. 5F). These indicated that FAIM3 was a target of miR‐344b‐5p.

Then, we assessed the functional roles of FAIM3 in miR-344b-5p-inhibited cells damage after IH exposure. As demonstrated in Fig. 5G–K, after co-transfecting miR‐344b‐5p mimics, pcDNA3.1-FAIM3 and corresponding negative control, overexpression of FAIM3 was able to markedly increase the suppressed cells proliferation while reducing promoted cells apoptosis caused by co-transfection of miR‐344b‐5p mimics when compared to negative control group. Taken together, these data indicated that FAIM3 could antagonize the effects of miR-344b-5p in IH-induced H9c2 cells injury.

### FAIM3 mediated XR_596701-associated cells damage in IH-induced H9c2 cells

**Fig. 6:**
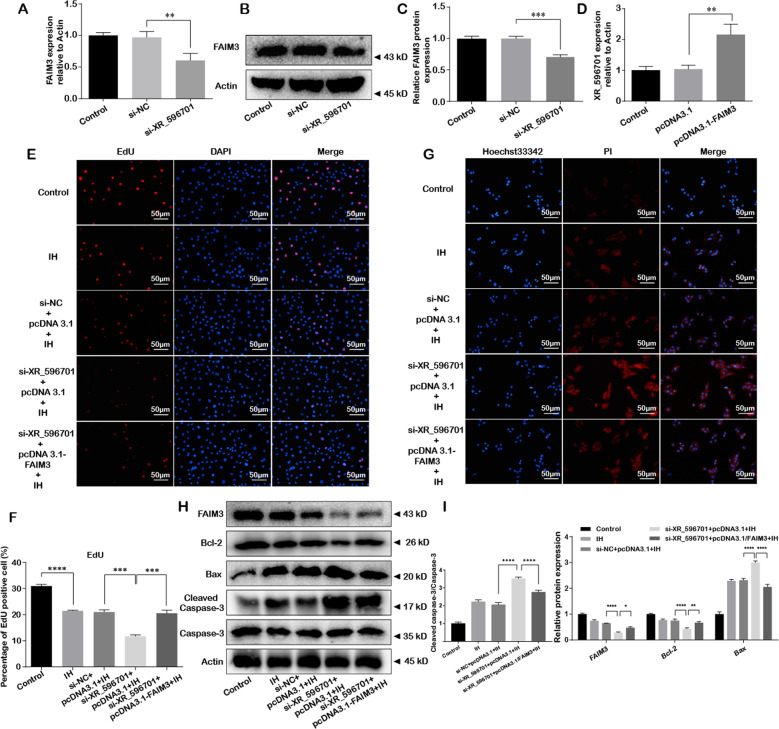
FAIM3 mediated XR_596701-associated cells damage in IH-induced H9c2 cells. **A** Relative FAIM3 expression was measure by qRT-PCR after transfecting si-XR_596701 or si-NC. **B**, **C** Relative FAIM3 protein expression was measure by western blot after transfecting si-XR_596701 or si-NC. **D** Relative XR_596701 expression was measure by qRT-PCR after transfecting pcDNA3.1 or pcDNA3.1-FAIM3. **E**, **F** si-XR_596701 (or si-NC) and pcDNA3.1-FAIM3 (or pcDNA3.1) were co-transfected into H9c2 cells. Cells were treated with IH for six cycles. EdU staining assay was performed to evaluate the proliferation of H9c2 cells, scale bar 50 µm. **G** Hoechst 33342/PI staining was performed to evaluate the apoptosis of H9c2 cells. si-XR_596701 (or si-NC) and pcDNA3.1-FAIM3 (or pcDNA3.1) were co-transfected into H9c2 cells, scale bar 50 µm. Cells were treated with IH for six cycles. **H**, **I** si-XR_596701 (or si-NC) and pcDNA3.1-FAIM3 (or pcDNA3.1) were co-transfected into H9c2 cells. Cells were treated with IH for six cycles. Expression levels of FAIM3 and apoptosis-related proteins (Bcl-2, Bax, and Cleaved caspase-3/Caspase-3) by western blot. *P < 0.05, **P < 0.001, ***P < 0.0001, ****P < 0.00001. Data were shown as Mean ± SD based on three independent experiments.

Considering that miR-344b-5p reversed the protective roles of XR_596701 and FAIM3 directly targeted miR-344b-5p expression in IH-induced cells injury, we assessed the potential relationship between FAIM3 and XR_596701 under IH stimulation. As shown in the Fig. 6A–D, the expressions of FAIM3 at mRNA and protein level were markedly decreased by si-XR_596701, while XR_596701 mRNA expression was significantly increased after transfecting pcDNA3.1-FAIM3. In addition, we found that FAIM3 expression was markedly upregulated after overexpression of XR_596701 (Fig. S4). Furthermore, by co-transfecting si-XR_596701, pcDNA3.1-FAIM3 and corresponding negative control before IH treatment of H9c2 cells, we found that overexpression of FAIM3 completely abolished the roles of XR_596701 on IH-mediated cells damage (Fig. 6E–I). These findings might explain that XR_596701 functions as a ceRNA to regulate the expression of FAIM3 by sponging miR‐344b‐5p.

## Discussion

**Fig. 7:**
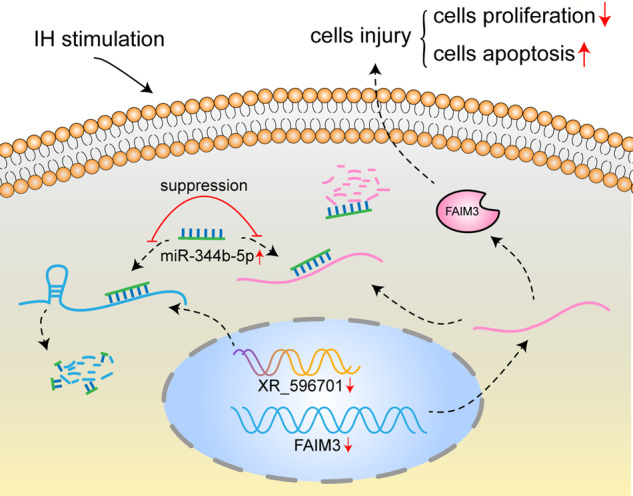
Proposed model for the pathological role and regulatory mechanism of XR_596701 in IH-mediated cardiomyocytes injury. Inhibition of XR_596701 increases the expression level of miR-344b-5p, which reduces the expression level of FAIM3, thus resulting in decreased proliferation and increased apoptosis in IH-induced H9c2 cells.

Cumulative evidence supports that OSA is an important risk factor for CV diseases, including myocardial infarction, hypertension and heart failure. IH is the common pathophysiological basis of OSA, which may exert critical role in OSA-associated CV diseases. Our previous study has showed that lncRNAs abnormally expressed in a rat model of IH and lncRNA XR_596701 was markedly increased in the IH-induced rat myocardial tissue. In this study, we performed a vitro model of IH-mediated cardiomyocytes injury to evaluate biological function and molecular mechanism of XR_596701. The results demonstrated that XR_596701 was upregulated and played a critical role in IH-induced H9c2 cells injury. In addition, we found that miR-344b-5p was verified to be a direct target of XR_596701 and miR-344b-5p inhibitor reversed the protective effects of XR_596701. Furthermore, FAIM3, a confirmed target of miR-344b-5p, exerted antagonistic effects in miR-344b-5p mediated IH-induced cells damage and affected protective effects of XR_596701 (Fig. 7).

lncRNAs are a class of non-coding RNA with the length of over 200 nucleotides. They can be divided into several groups and exert critical roles in modulation of gene expressions and biological processes at chromosome, transcription, and post-transcription, including chromosomal activation, cell proliferation, apoptosis, and death according to their length, localization, and genetic location [21, 22]. Recent studies are getting more and more involved relationship between lncRNAs and hypoxia. lncRNAs may regulate cancer proliferation, apoptosis, angiogenesis, metabolism and metastasis in hypoxic microenvironment [22]. For instance, Dr. Mole et al. showed that down-regulation of NEAT1 markedly suppressed the cell proliferation under hypoxic conditions in breast cancer [23]. Yang et al. demonstrated that histone deacetylase 3 may repressed the expression of lncRNA-LET via decreasing regulation of the lncRNA-LET promoter region [24]. Additionally, lncRNAs also exert an important role in hypoxia/reoxygenation (H/R) model of myocardial, hepatic, and cerebral injury [25–27]. Li et al. found that the expression of lncRNA XIST was markedly up-regulated and suppression of XIST could improve myocardial H/R injury through regulation of the miR-133a/SOCS2 axis [25]. In our study, lncRNA XR_596701 was significantly upregulated after IH stimulation, which was similar with the model of H/R, and played a protective role in IH-induced H9c2 cells injury. Nevertheless, how it functioned and what the underlying regulatory mechanism was is not clear.

MicroRNAs (miRNAs) are a class of important short strand RNA molecules that were first discovered in 1993 [28]. Previous review showed that a number of hypoxia-mediated miRNAs that have been implicated in pathogenesis of cardiovascular diseases modulated by hypoxia or HIF1α [29]. For example, miR-210, a hypoxia-induced miRNA, was significantly upregulated under hypoxia condition and played a protective effect in cardiovascular system by regulating cell proliferation, inhibiting apoptosis, and promoting angiogenesis [30]. Additionally, miR-885 was inhibited by H/R stimulation and overexpression of miR-885 could attenuate H/R-induced cell apoptosis by suppressing PTEN and BLC2L11 and regulating AKT/mTOR signaling [31]. Similarly, our current study also found that miR-344b-5p was noticeably decreased by IH treatment and rescued the H9c2 cells from IH-induced after overexpression of miR-344b-5p, which was consistent with protective functions of lncRNA XR_596701. Hence, we speculate that there is a interaction between miR-344b-5p and lncRNA XR_596701.

In recent years, several studies have found that lncRNAs may act as an ceRNA to regulate the expression and function of miRNA, which in turn bind to lncRNA, regulating gene expression and cellular function [32, 33]. According to Feng’s study, lncRNA DCRF was significantly increased after high glucose stimulation and act as a ceRNA to regulate cardiomyocytes autophagy by sponging miR-551b-5p [34]. Moreover, suppression of lncRNA NRF could antagonize RIPK1/RIPK3-mediated necrosis in cardiomyocytes and reduce necrosis and myocardial infarction upon ischemia/reperfusion (I/R) injury in the animal model by targeting miR-873 [35]. Interestingly, similar results were observed in our search. Initially, we found that XR_596701 was predominantly located in the cytoplasm by FISH assay, which indicated the possibility that XR_596701 functions as a miR-344b-5p sponge. Subsequently, we performed the luciferase reporter assay and RT-qPCR. The results validated that miR-344b-5p was a direct target of XR_596701. And further research demonstrated that downregulation of miR-344b-5p could reversed the protective effects of XR_596701 in IH-induced H9c2 cells injury. These findings deeply reveal the potential mechanism of protective functions of XR_596701.

As we all known, the effect of lncRNA-miRNA-mRNA axis in pathogenesis of CV diseases has been recently reported and highlighted. For instance, overexpression of lncRNA H19 up-regulated of miR-675 expression and H19-miR-675 axis played an important role in cardiac hypertrophy by targeting CaMKIIδ [36]. Furthermore, Wang et al. found that TUG1 served as an endogenous miR-9a-5p sponge and mediated HO-induced cardiomyocyte apoptosis that affected myocardial infarction progression through regulating miR-9a-5p/KLF5 axis [37]. In our study, we have validated the interaction between XR_596701 and miR-344b-5p, but their further potential mechanism in IH-induced H9c2 cells injury is unknown. Thus, we hypothesized that XR_596701 plays protective effect on IH-mediated H9c2 cells damage through lncRNA-miRNA-mRNA axis. FAIM3, also called IgM-Fc receptor (FcμR), was originally designated as an inhibitor of Fas/CD95-induced apoptosis and has been reported to be related to innate immune responses, IgM binding and apoptosis [38]. Richte et al. showed that down-regulation of FAIM3 markedly increased susceptibility to apoptosis of activated T cells and presumed that FAIM3 exerts function in the regulation of activation-induced cell death (AICD) [39]. We predicted FAIM3 may be a potential target of miR-344b-5p by bioinformatics analysis. And the results of luciferase reporter assay and RT-qPCR confirmed that FAIM3 was a target of miR‐344b‐5p. Subsequently, we performed gain and loss of function approaches, the results indicated that FAIM3 could resist the effects of miR-344b-5p and abolish the protective roles of XR_596701 on IH-mediated cells damage. These findings reveal that XR_596701 play protective roles on IH-induced cells injury via miR‐344b‐5p/ FAIM3 axis.

The aim of the present study was only to evaluate the roles and underlying mechanism of XR_596701 in vitro model just as a preliminary investigation. We acknowledge, however, that this study has several limitations. Primarily, we only investigated the effect and molecular mechanism of XR_596701 in vitro. A well performed characterization of XR_596701 downregulated or knock-out mice models would greatly improve completeness of the study. In the future, we will establish animal models based on XR_596701 to verify our current research results. Additionally, although our research may help to better understand the pathogenesis of myocardial IH-induced damage, how to better use it in the treatment of OSA-induced CV patients needs further investigations in clinical settings. Moreover, XR_596701 may be involved in multiple signaling pathways, which play an important role in the regulation of IH-induced H9c2 cells damage. Therefore, we will focus on the effects of other target genes of XR_596701 on IH-induced H9c2 cells injury in future studies.

## Conclusion

In conclusion, our findings offer insight into XR_596701-miR-344b-5p-FAIM3 axis mediated IH-induced H9c2 cells injury and suggest that XR_596701 might be used as a new biomarker and therapeutic target for OSA patients with CV diseases.

## Materials and methods

### Cell culture and IH injury model

Rat cardiomyocyte‐derived H9c2 cells (ATCC, Shanghai, China; The STR profiling of cell line was showed in the supplementary file) were cultured at 37 °C in 5% CO_2_ atmosphere with Dulbecco’s modified Eagle’s medium (HyClone), which contained 10% fetal bovine serum (Gibco) and 1% penicillin/streptomycin. IH model of H9c2 cells was established as described previously [18]. Briefly, H9c2 cells were transferred to hypoxic chamber with 1% O_2_ at 37 °C for 35 min; then, the cells were incubated with normoxic chamber with 21% O_2_ at 37 °C for 25 min. Cell maintained repeated IH stimulation for six cycles.

### LncRNA identification

Full-length XR_596701 cDNA was obtained by 5′ and 3′ rapid amplification of cDNA ends (RACE). Subsequently, to identify the characteristic of XR_596701, genome location and protein-coding potential were analyzed by NCBI Genome, ORF Finder (https://blast.ncbi.nlm.nih.gov/Blast.cgi) and the Coding Potential Calculator (CPC) [40], respectively. ORF finder is a graphical analysis tool, which can search newly sequenced DNA for potential protein encoding fragment and then validate predicted protein by Smart BLAST. Eventually, we combined above results to assess the molecular characteristics of lncRNA.

### RNA fluorescent in situ hybridization (RNA-FISH)

DIG-labeled lncRNA XR_596701 probes were obtained from Servicebio (Wuhan, Chian). H9c2 cells were fixed in 4% paraformaldehyde for 20 min at room temperature (RT). After three time washed in PBS, cells were permeabilized with 0.5% Trixon-100 for 5 min. Then, the cells were blocked by prehybridization solution for 30 min at 37 °C. After removal of the prehybridization solution, the cells were cultured with the probe hybridization solution containing specific probe overnight in the dark. Finally, the cells were incubated with DAPI for 15 min, and observed using a laser confocal microscope (Leica SP5, Heidelberg, Germany). The sequence for XR_596701 probe is: 5′-DIG-GAGGTCATAAGGAAGTTTGGACACCGCAGAA-DIG-3′.

### Quantitative real-time polymerase chain reaction (qRT-PCR) analysis

Total RNA from H9c2 cells was extracted using a Trizol reagent (Takara) and cDNA was synthesized using a Reverse Transcription Kit (#K1622; Thermo Fisher Scientific). Real-time fluorescence quantification PCR was conducted with a SYBR Green Kit (#K0223; Thermo Fisher Scientific). The relative expressions of lncRNA and miRNA were calculated based on Ct values and β-actin and U6 were served as controls. The qRT-PCR was detected on an ABI 7500 thermocycler (Applied Biosystems, Foster City, CA, USA). All PCR primers are listed in Table S1.

### Cell transfection

Small interfering RNA (siRNA) for XR_596701 (si-XR_596701), negative control si-NC, miR‐344b-5p mimics, miR‐344b-5p inhibitor and corresponding negative control (mimics NC or inhibitor NC) were obtained from Gene Pharma company (Shanghai, China). XR_596701 overexpression vectors (oe-XR_596701) and FAIM3 overexpression plasmid pcDNA3.1-FAIM3 and negative control pcDNA3.1 were constructed by Han heng biotechnology company (Shanghai, China). The transfection of H9c2 cells was performed by using Lipofectamine® 3000 (Invitrogen, Thermo Fisher Scientific). At 6 h of post-transfection, culture media were replaced with serum-free medium and cells were continued to incubate for another 48 h.

### Immunofluorescence (IF)

H9c2 cells were fixed in 4% paraformaldehyde for 15 min at RT and permeabilized with 0.5% TritonX-100 for 5 min. After permeabilization, the cells were blocked by incubation with 10% FBS at RT for 1 h. Then, H9c2 cells were incubated with anti-Caspase-3 antibody (1:50, Santa Cruz) at 4 °C overnight. Next, the cells were washed three times with PBS and incubated with secondary anti-mouse FITC (diluted 1:100) in the dark at RT for 1 h. Nuclei were stained for 10 min with DAPI. After three times washes in PBS, coverslips were mounted on slides with anti-fade mounting medium and evaluated under a laser confocal microscope (Carl Zeiss, LSM 510).

### 5-ethynyl-2′-deoxyuridine (EdU) staining

The proliferation of H9c2 cells was detected by BeyoClick™ EdU Cell Proliferation Kit with Alexa Fluor 594 following the manufacturer’s instructions. In brief, H9c2 cells were seeded in 12-well plates and incubated with 100 µl of 50 µM EdU for 2 h after IH stimulation. And then the cells were washed three times with PBS, fixed with 4% paraformaldehyde for 15 min, and permeabilized with 0.5% Trixon-100 for 5 min. According to the kit, the cells were incubated in the mixture of Click Reaction Buffer, CuSO4, Azide 594, and Click Additive Solution for 30 min at RT. After three times washes in PBS, the cell nucleus was stained with 100 µl DAPI for 20 min. The staining cells were observed using a fluorescence microscope (Olympus, CKX41-F32FL, Japan).

### Hoechst 33342/PI staining assay

The apoptosis of H9c2 cells was measured by Hoechst 33342/PI staining Kit (GENVIEW) following the manufacturer’s instructions. H9c2 cells were harvested and washed three times with PBS after IH stimulation. Then the cells were stained by incubating in staining buffered solution containing 500 nM Hoechst 33342 and 500 nM PI at RT for 15 min in the dark. Finally, they were washed with PBS three times and observed under a fluorescence microscope (Olympus, CKX41-F32FL, Japan).

### Flow cytometry

H9c2 cells apoptosis was detected via flow cytometry using the Annexin V/PI kit (Biyuntian, China). Briefly, H9c2 cells were seeded at 1 × 10^5^ cells/well and resuspended in 1× binding buffer. Subsequently, cells were stained with 5 *μ*l of Annexin V-FITC and 10 μl of PI. Finally, apoptotic cells were analyzed by a flow cytometer (Becton Dickinson, USA).

### Dual-luciferase reporter assay

The wild‐type or mutant XR_596701 and FAIM3 was inserted into the Xho1 and Not1 sites of pSI-Check2 luciferase reporter vector. Cells were transfected with pSI-Check2‐XR_596701/FCMR‐wt or pSI-Check2‐XR_596701/FAIM3‐mut and miR‐344b‐5p mimics using Lipofectamine® 3000. The luciferase activity was measured after transfection by utilizing the Dual-Luciferase Reporter Assay kit (Promega) and analyzed it with a luciferase reporter assay system (Promega). Luciferase activity was normalized to renilla luciferase activities.

### Western blot assay

H9c2 cells were lysed using Mammalian Protein Extraction Reagent (CWBIO, Beijing, China) and removed the cells debris through centrifuging for 15 min (15,000 rpm, 4 °C). Total protein was harvested and quantified using BCA assay kit (DINGGUO, Beijing, China). For each sample, approximately 20 μg proteins was separated by SDS-PAGE and transferred to a PVDF membrane. Then PVDF membrane was blocked in 5% non-fat dry milk for 2 h, followed by incubation with primary antibodies against Caspase-3 (1:1000 dilution, #14220, Cell Signaling Technology), Bcl-2 (1:1000 dilution, #3498, Cell Signaling Technology), Bax (1:5000 dilution, ab32503, Abcam), FAIM3 (1:1000 dilution, A6320, Abclonal, China), β-actin (1:1000 dilution; #4970, Cell Signaling Technology) at 4 °C overnight. Subsequently, the membrane was incubated with a secondary antibody (1:5000) at RT for 1 h. After washes, the immunoreactivity was detected using the standard chemiluminescence (Thermo-Fisher). The bands were quantified by measuring the band intensity for each group.

### Statistical analysis

The SPSS 19 software and GraphPad Prism 8 software were used to carry out statistical analysis. All quantitative data are expressed as mean ± SD from at least three independent experiments. The t-test was performed for comparisons between two groups and one-way analysis of variance (ANOVA) followed by Tukey’s post hoc test was used for comparisons among multiple groups. The *P* value less than 0.05 was considered statistically significant.

## Supplementary information

Supplementary material

Supplementary material-H9c2 cells STR Profiling Report

## Data Availability

The data that support the findings of this study are available from the corresponding author upon reasonable request.
