# Supplementary material for: LncRNA XR_596701 protects H9c2 cells against intermittent hypoxia-induced injury through regulation of the miR-344b-5p/FAIM3 axis
Source: Cell Death Discov. 2022 Jan 28;8:42. doi: 10.1038/s41420-022-00834-8 (PMC8799738; doi:10.1038/s41420-022-00834-8)
Supplement: Supplementary file 1 — Supplementary material [file 41420_2022_834_MOESM1_ESM.docx]

| Table S1. Sequence information | |
| --- | --- |
|  | Sequence (5’-3’) |
| XR_596701 | Sense: CCAGTTTACAGAGATTCCTCAGG  Antisense: GCCAAGTGACATGAACAAAGTG |
| si-XR_596701 | Antisense: CCAGCUGGAAUGGAGUCUUTT |
|  | Sense: AAGACUCCAUUCCAGCUGGTT |
| special stem-loop primer  of miR-344b-5p | GTCGTATCCAGTGCGTGTCGTGGAGTCGGC  -AATTGCACTGGATACGACGAATATA |
| miR-344b-5p | Sense: GGAGTCAGGCTGCTGGTTA |
|  | Antisense: CAGTGCGTGTCGTGGAGT |
| miR-344b-5p mimics | Sense: AGUCAGGCUGCUGGUUAUAUUC |
|  | Antisense: GAAUAUAACCAGCAGCCUGACU |
| mimics NC | Sense: UUGUACUACACAAAAGUACUG |
|  | Antisense: GUACUUUUGUGUAGUACAAUU |
| miR-344b-5p inhibitor | GAAUAUAACCAGCAGCCUGACU |
| inhibitor NC | CAGUACUUUUGUGUAGUACAA |
| FAIM3 | Sense: CAGCCACCACACCAGACTTCATG |
|  | Antisense: CTCGCATTCTCCTCGCCATTCG |
| si-FAIM3 | Sense: CCAACAUCUUUGUCAAGAATT |
|  | Antisense: UUCUUGACAAAGAUGUUGGTT |
| U6 | Sense: CTCGCTTCGGCAGCACA |
|  | Antisense: AACGCTTCACGAATTTGCGT |
| β-actin | Sense:CGAGTACAACCTTCTTGCAGC |
|  | Antisense:ACCCATACCCACCATCACAC |

| Table S2. The full-length nucleotide sequence of XR_596701 |
| --- |
| 1 CAGGGCTTTT GAACAGTACT TGAATCTGGA AGACAGTAGG CTGCTGAGTC ATCTGAAGAC  61 GTGTTCTGCG GTGTCCAAAC TTCCTTATGA CCTCTGGTTC AAAAGGTGCT TCGCGGGATG  121 CCTACCCGAG TCCAGTTTAC AGAGATTCCT CAGGACAGCT CAGATGCCAT CGTGAGCAAG  181 GCCATCGACT TGTGGCACAA ACACTGTGGG ACCCCAGTCC ATTCAGCCTG ACAGCTCGTG  241 ACTTCATGGA CAGCCTGCAG ACCCTCCCTG AGCACTTTGT TCATGTCACT TGGCCTACTG  301 ACTGGTTAGA AATAACAGTC TTGCTGTGGT GCTCCAAATG CAACACCCCT CCCCCAGTCA  361 GTAAAATAAT TTAATCAAGA TGCCTGGCCT GGCCACGTTA TGAAGCCGCA TCTCTTAGTG  421 ACACTGACAT GCAACTCTGG GCTAGTATTC TAAAAGGGTG ACTAGGAAAT TCCTCCGGGT  481 CTGGGCAGCT CTGTCAGGGC AGCCCGGGCA CTCTTGTGGA TGGTAAGAGT GAAGCCCTGC  541 CCCCTCTGAT GACCACCAAG ACACACAGTT ACTTAGGAAG GCCTGGTTCT GGAAGAAGAG  601 CAGCTTTCTT TTCCTGGCCT CTCTGATTTG GCTTTAGCCT TCTGTTGCCC TAGGACGTGT  661 GTAAGTGCGA GCTGACTGCT GTCTCATGCC CTGCCTGACT AAACTGGCTT AAAGACAGTC  721 CCTGCATTTC CTTACATGTT CCCTCCTTTG TGCCAGCTGG AATGGAGTCT TGTCTTTTCC  781 TTCCTGAGAC AAAGCTTCAT TCTGTACCTC ACCTTTCTGA AGCTCTTTAT GTAGCTGGCC  841 TTGAAGTCCT AGCTGTCCTC CTGCCTCAGC CTCCTGAGTG CTGGGATTTT ATTAAATAAA  901 ATACCTCTAA ATTTACCTTC TAGGAGAAGG AGAAAGCA |

| Table S3. ORF Finder of XR_596701 sequence | | | | | |
| --- | --- | --- | --- | --- | --- |
| [**Label**](https://www.ncbi.nlm.nih.gov/orffinder) | [**Strand**](https://www.ncbi.nlm.nih.gov/orffinder) | [**Frame**](https://www.ncbi.nlm.nih.gov/orffinder) | [**Range**](https://www.ncbi.nlm.nih.gov/orffinder) | [**Length (nt \| aa)**](https://www.ncbi.nlm.nih.gov/orffinder) | **SmartBLAST** |
| ORF3 | + | 3 | 87-374 | 288 \| 95 | Not significant similarity found |
| ORF5 | + | 3 | 762-899 | 138 \| 45 | Not significant similarity found |
| ORF6 | - | 1 | 150-284 | 135 \| 44 | Not significant similarity found |
| ORF1 | + | 1 | 118-231 | 114 \| 37 | Tbc1d7 |
| ORF8 | - | 2 | 137-247 | 111 \| 36 | Not significant similarity found |
| ORF2 | + | 1 | 736-834 | 99 \| 32 | Not significant similarity found |
| ORF4 | + | 3 | 549-626 | 78 \| 25 | Not significant similarity found |
| ORF7 | - | 2 | 650-727 | 78 \| 25 | Not significant similarity found |

| Table S4. CPC values predicted for XR_596701 | | | | | | |
| --- | --- | --- | --- | --- | --- | --- |
| **ID** | **Label** | **Coding probability** | **Peptide length(aa)** | **Fickett score** | **Isoelectric point** | **ORF integrity** |
| **XR_596701** | **Non coding** | **0.190916** | **96** | **0.3629** | **6.88629150391** | **complete** |


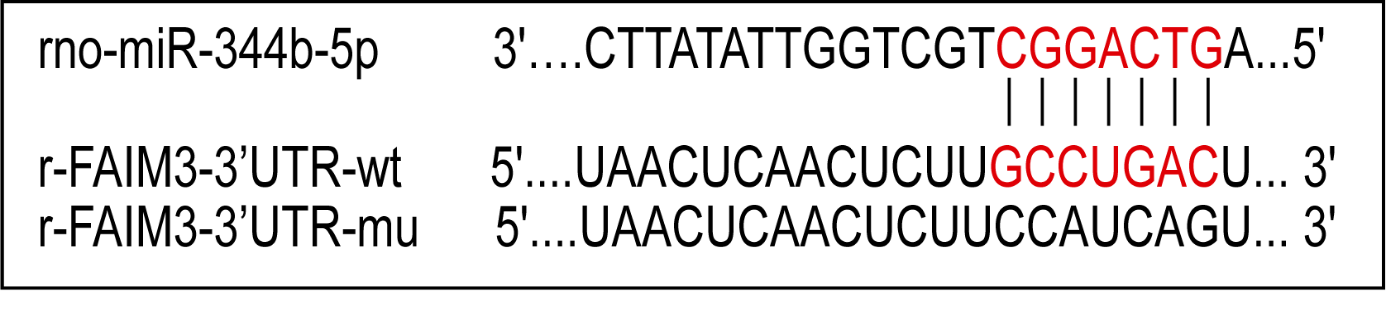


Figure S1. The putative binding site between miR-344b-5p and FAIM3.


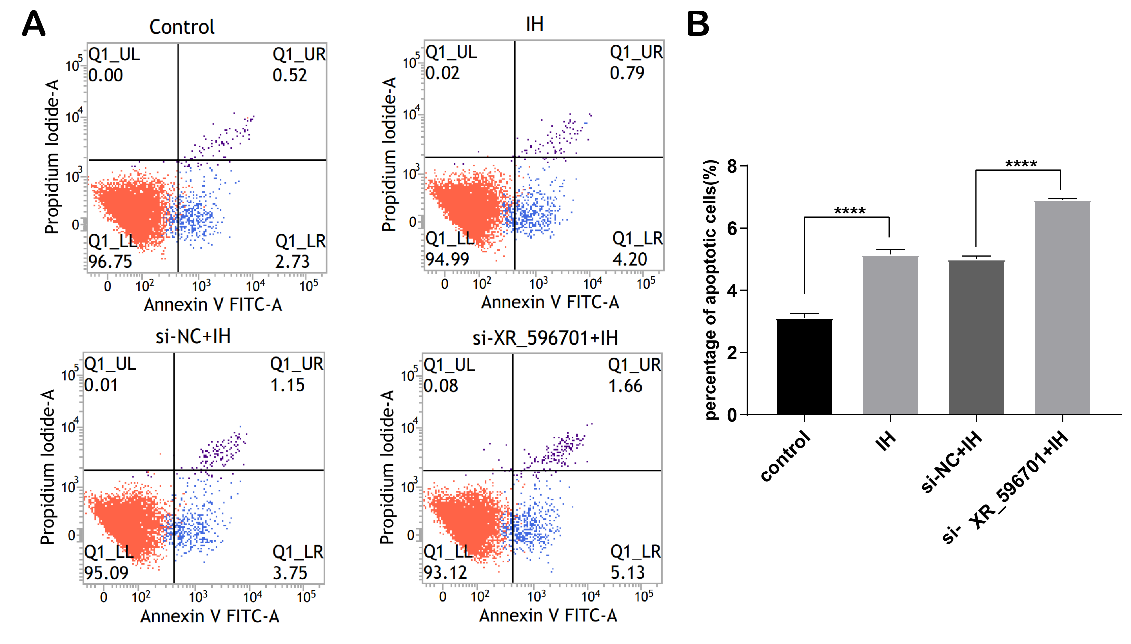


Figure S2. H9c2 cells apoptosis was detected by flow cytometry.


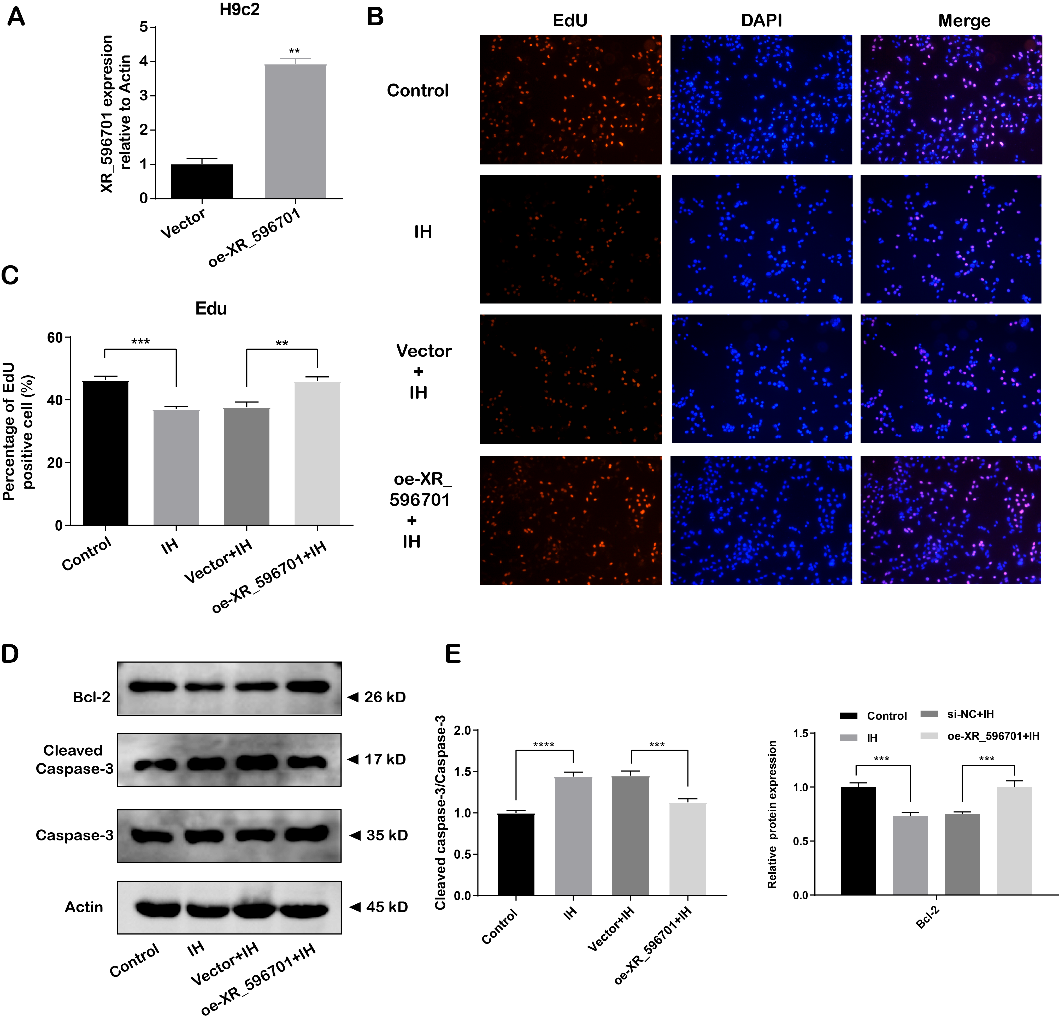


Figure S3. The potential functional effects of XR_596701 in IH-induced H9c2 cells after overexpression of XR_596701. (A) H9c2 cells were transfected with overexpression plasmid of XR_596701 or empty vector. Relative XR_596701 expression was measure by qRT-PCR. (B-C) EdU assay was performed to assess the impact of XR_596701 on the proliferation of H9c2 cells. Red (EdU) H9c2 cells indicated proliferating the cell nucleus and blue (DAPI) represented the cell nucleus, scale bar 50 µm. (D-E) Expression levels of apoptosis-related proteins (Bcl-2 and Cleaved caspase-3/Caspase-3) by western blot analysis. *P < 0.05, **P < .001, ***P < .0001, ****P < .00001. Data were shown as Mean ± SD based on three independent experiments.


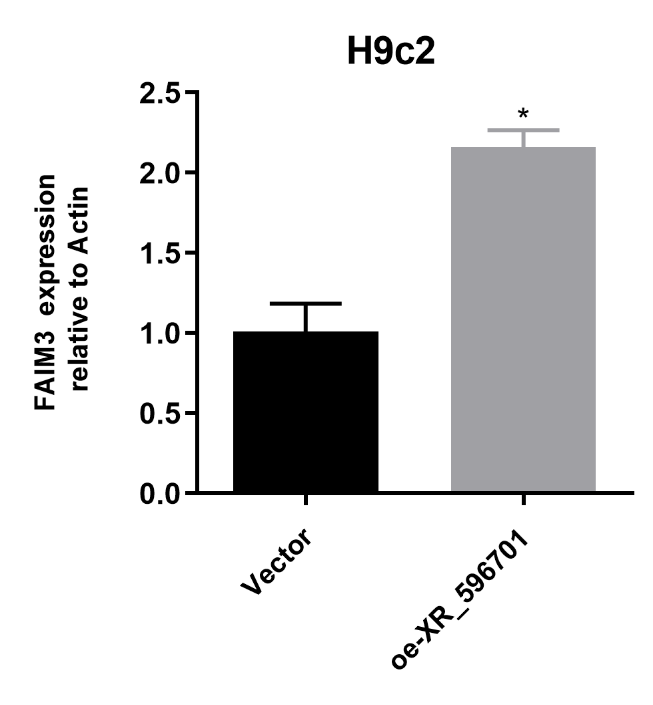


Figure S4. Relative FAIM3 expression was measure by qRT-PCR after transfecting with overexpression plasmid of XR_596701 or empty vector.
