# Supplementary material for: LncRNA XR_596701 protects H9c2 cells against intermittent hypoxia-induced injury through regulation of the miR-344b-5p/FAIM3 axis
Source: Cell Death Discov. 2022 Jan 28;8:42. doi: 10.1038/s41420-022-00834-8 (PMC8799738; doi:10.1038/s41420-022-00834-8)

Cell Line Authentication Service

STR Profiling Report

Sample Type: Cell Line

Sample from: FuHeng Cell Center，Shanghai，China

Testing Method: STR Genotyping

Report Time: June 09, 2020.

Cell Line Authentication – STR Profiling Report

Sample code

Table 1. Sample Code

Customer's code	Company Code
H9c2	20200604-01

Sample Number:1

Sample Type: Cell line

Testing Type: STR

Sample From: FuHeng Cell Center，Shanghai，China

Testing Method:

DNA was extracted by a commercial kit from CORNING (AP-EMN-BL-GDNA-250G). The ten STRs including one human locus were amplified by multiplex PCR and separated on ABI 3730XL Genetic Analyzer. The signals were then analyzed by the software GeneMapper.

Data Interpretation:

Cell lines were authenticated using Short Tandem Repeat (STR) analysis as described in 2012 in ANSI Standard (ASN-0002) by the ATCC Standards Development Organization (SDO) and, mouse cell line authentication cytotechnology.2014;66:133-147.

Test Results:

1. Result

Table 2. Matching information on the cell lines

Sample Code	Multi-allele	Cell line matched	Cross contamination	EV
20200604-01	No	No match	No	-

- Multi-allele means some STR contain more than two loci.

2. Sample Description

A. The STR results showed that the DNA quality control of H9c2 cells is no problem. PCR detected DNA typing. As a result, no one of 21 sites was found in the cell line. At the same time, in the mixed strategy of human and mouse, there was no result of murine origin. It is concluded that the sample excludes sources of human and mouse cells.

B. No cross contamination was found in the cell line, and the cell line was normal. As the STR database was not logged in, the matching result could not be displayed. If the paper was to publish, these data could be submitted to the magazine.

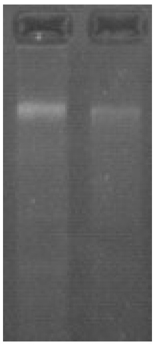

On the left is the tested DNA, on the right is the positive control

Others:

1. Genotyping Strategy and Site Distribution

Attached Table. Experimental Strategy and Sites

	Panel1	Panel2	Panel3	Panel4
1	D3S1358	D8S1179	D19S433	AMEL
2	VWA	D21S11	TH01	D1S1656
3	D7S820	D16S539	D13S317	D5S818
4	CSF1PO	D2S1338	TPOX	D12S391
5	PENTAE	PENTAD	D18S51	FGA
6			D6S1043	

The allele match algorithm compares the 8 core loci plus amelogenin only, even though alleles from all loci will be reported when available.

2. DSMZ tools was used to carry on the cell line comparison, which contains 2455 cell lines STR data from ATCC, DSMZ, JCRB ,ECACC， GNE and RIKEN databases. If the cell is not included in the above cell library, users need to compared with other databases.

20200604SHU

Sample File	Sample Name	Panel	OS	SQ
63_G08_CellLineAuthentication-1--0605.fsa	H9C2	Xiao shu STR-1		

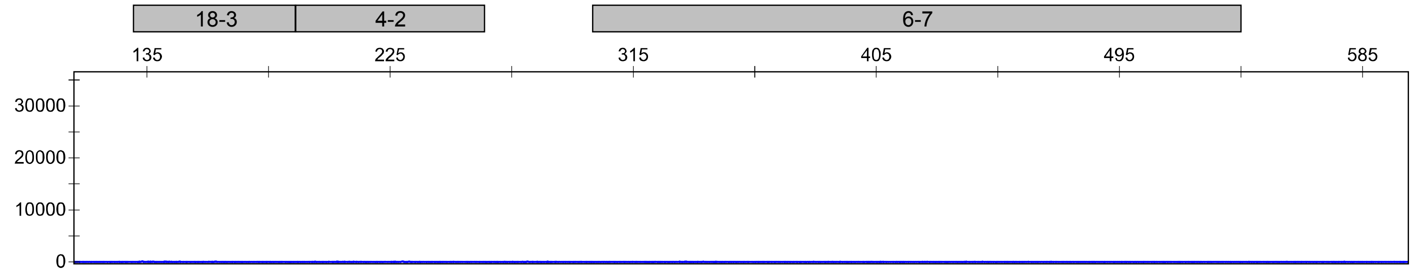

63_G08_CellLineAuthentication-1--0605.fsa	H9C2	Xiao shu STR-1		

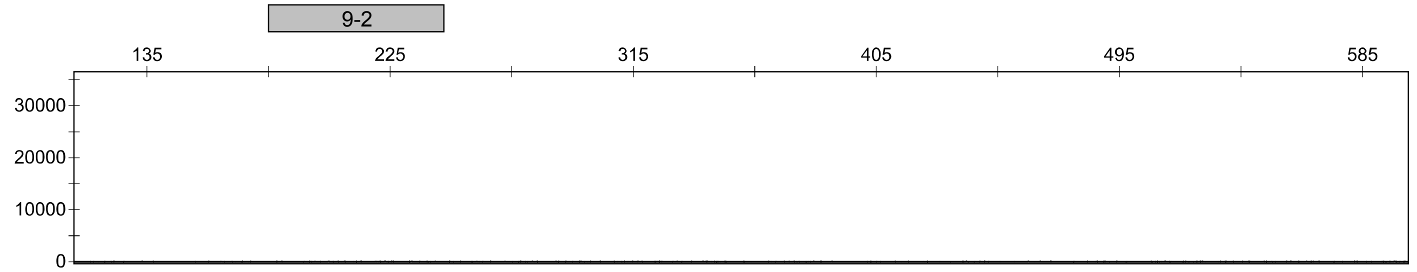

79_G10_CellLineAuthentication-1--0605.fsa	H9C2	Xiao shu STR-2		

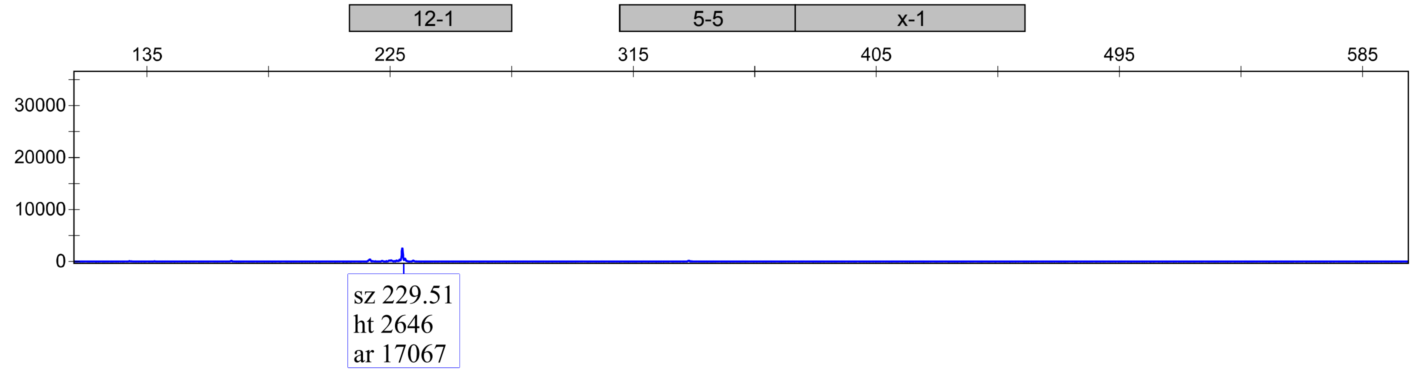

79_G10_CellLineAuthentication-1--0605.fsa	H9C2	Xiao shu STR-2		

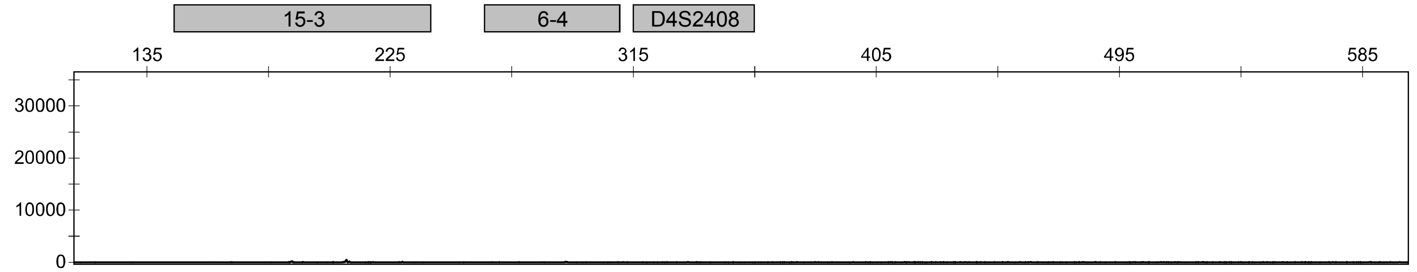

Supplement: Supplementary file 2 — Supplementary material-H9c2 cells STR Profiling Report [file 41420_2022_834_MOESM2_ESM.pdf]
